# Pulp Therapies Rooted in Evidence: IAPD Porto Declaration

**DOI:** 10.1111/ipd.70073

**Published:** 2026-01-14

**Authors:** Vineet Dhar, Ikhlas El‐Karim, James A. Coll, Ashraf F. Fouad, Anne C. O'Connell, Saeed Asgary, Lars Bjørndal, Zafer C. Cehreli, Yasmi O. Crystal, Manikandan Ekambaram, Brian D. Hodgson, Nicola P. Innes, Jonas Almeida Rodrigues, Nessrin A. Taha, Nitesh Tewari, Tugba Turk

**Affiliations:** ^1^ Department of Orthodontics and Pediatric Dentistry University of Maryland, School of Dentistry Baltimore Maryland USA; ^2^ School of Medicine Dentistry and Biomedical Sciences Queen's University Belfast Belfast UK; ^3^ The Department of Endodontics School of Dentistry at University of Alabama at Birmingham Birmingham Alabama USA; ^4^ Division of Public and Child Dental Health Dublin Dental University Hospital, Trinity College Dublin Ireland; ^5^ Iranian Center for Endodontic Research, Research Institute of Dental Sciences Shahid Beheshti University of Medical Sciences Tehran Iran; ^6^ Cariology and Endodontics, Section of Clinical Oral Microbiology, Department of Odontology, Faculty of Health and Medical Sciences University of Copenhagen Copenhagen Denmark; ^7^ Department of Pediatric Dentistry, Faculty of Dentistry Hacettepe University Ankara Turkey; ^8^ Department of Pediatric Dentistry New York University. NY New York City New York USA; ^9^ Paediatric Dentistry Discipline, Department of Oral Sciences, Faculty of Dentistry University of Otago Dunedin New Zealand; ^10^ Marquette University School of Dentistry Milwaukee Wisconsin USA; ^11^ School of Dentistry College of Biomedical and Life Sciences Cardiff University Cardiff UK; ^12^ Universidade Federal do Rio Grande do Sul (UFRGS) Porto Alegre Brazil; ^13^ Department of Conservative Dentistry, Faculty of Dentistry Jordan University of Science and Technology Irbid Jordan; ^14^ Pediatric and Preventive Dentistry, Centre for Dental Education and Research All India Institute of Medical Sciences New Delhi India; ^15^ Ege University, School of Dentistry Department of Endodontics Izmir Turkey

**Keywords:** endodontics, pulp biology/basic sciences, traumatic injuries

Recent advances in our understanding of pulpal biology and inflammatory responses have led to a fundamental shift in our approach to pulpal and periapical diseases. The traditional dichotomy of reversible versus irreversible pulpitis has been challenged by evidence suggesting that pulpal inflammation exists on a continuum, rather than as discrete states [1, 2]. This continuum includes initial, mild, moderate and severe pulpitis [1]. Pulpitis may also progress apically in a manner that allows incremental removal of the affected portion of the inflamed pulp, which can help alleviate the accompanying severe symptoms. Pulpal inflammation represents a complex defensive response that can result in either tissue healing or degeneration and necrosis if left untreated. While clinical symptoms and radiographic signs and pulp sensibility tests provide guidance, they do not accurately indicate the healing potential of the inflamed pulp. In addition, they often correlate poorly with the actual histological status of the pulp [3]. This biological reality, coupled with improved bioactive materials, demands a reconsideration of conventional treatment approaches. These considerations include diagnostic classifications that more accurately reflect the biological continuum of pulpal disease, treatment decisions based on objective clinical findings, protocols that prioritise pulp preservation when biologically feasible, and long‐term outcome assessments that validate contemporary methods. The translation of these scientific advances to clinical practice requires updated evidence‐based guidelines that can inform decision‐making while acknowledging the complexity of pulpal biological responses.

The methodology for developing the International Association of Paediatric Dentistry (IAPD) Global Consensus Statement on pulp therapies in primary and permanent teeth involved a systematic consensus‐building process with 16 global experts in paediatric dentistry, endodontics, and cariology who participated in the 3rd IAPD Summit held in Porto, Portugal, in November 2024. The process commenced with pre‐summit activities, during which experts were organised into four working groups, each focusing on distinct aspects of pulp therapy. Each expert prepared evidence‐based drafts following predetermined search strategies. The key conclusions from their papers were consolidated into recommendation statements for primary and permanent teeth. The consensus development utilised a Delphi process with statements requiring over 70% agreement. The process led to formulation of consensus‐based recommendations and consensus‐based statements. The consensus‐based recommendations were further categorised into two types: strong recommendations based on evidence from randomised controlled trials, systematic reviews, meta‐analyses, or clinical practice guidelines, and weak or conditional recommendations based on lower‐quality studies. The consensus‐based statements reflected the expert opinion of the panel and Delphi agreement was documented.

Working Group 1 established the fundamental principles for pulp diagnosis and caries management, emphasising assessment methods and conservative approaches to caries removal. Working Group 2 focused on vital and non‐vital pulp therapy techniques in primary teeth. Working Group 3 provided guidance for the treatment of permanent teeth, specifically regarding the pulp therapy approaches for various clinical scenarios. Working Group 4 outlined protocols for managing traumatic dental injuries, addressing both immediate care and long‐term complications. This paper summarises the key findings of the IAPD Porto Summit on evidence‐based pulp therapies.

In teeth with deep caries diagnosed with normal or reversible pulpitis (as per American Association of Endodontics (AAE) terminology [4] or in accordance with Wolters' [1] initial or mild pulpitis criteria), the unexposed pulp, if provided a favourable environment, can heal through the production of reactionary dentine. For both primary and permanent teeth, the recommendations emphasise pulpal assessment, which combines clinical signs, symptoms, history and radiographic findings.

However, the workgroup identified a distinction in diagnostic testing—while cold and electric pulp testing shows high reliability in mature permanent teeth [5], diagnostic testing in paediatric patients should be performed on a case‐by‐case basis for primary teeth and immature permanent teeth [6, 7], with greater reliance on patient history as well as clinical and radiographic findings. The workgroup agreed that dental dam isolation, good illumination, and magnification were important to aid in pulpal diagnosis and improve treatment outcomes [8, 9].

A consistent theme across both primary and permanent teeth workgroups highlighted the evidence supporting conservative approaches, particularly selective caries removal over non‐selective (complete) caries removal [6, 9, 10], provided that the tooth is asymptomatic or mildly symptomatic, and normally responsive to pulp sensibility testing. The workgroups classified deep carious lesions as those extending into two‐thirds (inner third) to three‐fourths (inner quarter) of the dentine thickness, with a definitive radiographic barrier of dentine [9, 10]. These lesions can be categorised as ICDAS 5 lesions [11], are close to the pulp, and may be at risk for pulp exposure if carious tissue is removed non‐selectively [12]. A pragmatic radiographic threshold for deep caries in primary teeth was defined as two‐thirds (inner third) of dentine thickness, while in permanent teeth, it is three‐fourths (inner quarter) of dentine thickness, provided the tooth shows no signs of irreversible pulpitis (as per AAE [8] or in accordance with Wolters' [1] moderate or severe pulpitis criteria). For deep caries, clinicians may use magnification, such as a microscope, to assist in non‐selective tissue removal to reduce the chances of pulpal exposure. However, stepwise excavation, followed by durable temporary restoration (such as glass ionomer cement) and re‐entry in 8–10 weeks to allow formation of tertiary dentine, is considered a viable option with fewer pulp exposures for carious lesions that extend into the inner quarter of dentine [13]. At the re‐entry visit, non‐selective caries excavation is completed followed by a definitive restoration [13]. Nonetheless, selective removal to soft dentine followed by definitive restoration can be accomplished in a single appointment with minimal risk of pulp exposure [10] and has shown comparable outcomes [12]. This approach may be beneficial in paediatric patients, both for primary and permanent teeth.

For extremely deep caries lesions that extend beyond three‐fourths of the dentine thickness and show no radiographic evidence of a barrier of dentine between caries and pulp, the pulp may be infected and severely inflamed irrespective of symptoms. In such cases, non‐selective (complete) caries removal is the preferred approach, followed by a thorough examination and management of the pulp, which is likely to be exposed.

Research indicates that severe inflammation occurs typically when decay is very close to the pulp (< 0.5 mm) [14, 15]. Additionally, infection advancing to the pulp is not observed in teeth with normal or reversibly inflamed pulp [16]. Furthermore, the number of bacteria remaining in sealed carious lesions was not found to be higher than the number detected in dentine after non‐selective (complete) caries removal [17]. These findings support the practice of selective caries removal in permanent teeth with deep caries that have a normal pulp or signs of reversible pulpitis (as per AAE [8] or in accordance with Wolters' [1] initial or mild pulpitis criteria). This conservative and biological approach protects the pulp, aims to create a favourable environment for pulpal healing, minimises pulp exposures, can be completed in a single appointment, and is considered cost‐effective [18, 19].

In contrast to the traditional understanding that Indirect Pulp Treatment (IPT) involves the removal of most carious tissue, leaving behind just enough to prevent pulp exposure [20], recent clinical practice guidelines by the American Academy of Paediatric Dentistry (AAPD) highlighted a shift in this approach. The updated AAPD guideline recommends that IPT for primary and permanent teeth should involve a more conservative approach leaving deepest decay after selective caries removal to prevent pulp exposure, followed by the placement of a liner [21, 22].

In cases of small pulp exposures that occur during caries excavation in permanent teeth, direct pulp capping (DPC) can be performed, and evidence supports that calcium silicate cements (CSCs) should be the material of choice for the liner [9]. However, pulpotomy procedures have a better success rate than DPC in primary teeth [21].

For extremely deep caries lesions extending through the entire radiographic thickness of dentine, histological data shows that bacteria may be present in either tertiary dentine or within the pulp [23]. In such cases, direct inspection of pulp tissue is recommended prior to performing a partial or full pulpotomy procedure in mature permanent teeth, depending on the pulp status [21]. Evidence suggests that cases traditionally diagnosed as irreversible pulpitis can often be treated successfully with pulpotomy, indicating the feasibility of maintaining much of the pulp tissue [24]. For immature permanent teeth with extremely deep caries, pulpotomy (partial or full) is recommended to support root development [25]. Bioactive materials, particularly CSCs, are the preferred material across both dentitions for vital pulp therapy procedures [9].

A full pulpotomy is also recommended in primary teeth with extremely deep caries and history of spontaneous pain but no signs of necrosis, provided that haemostasis can be obtained. At present, there is a lack of data on the success of partial pulpotomies in primary teeth [21, 26].

The workgroup acknowledged the unique healing capacity of dental pulp following trauma, with particular emphasis on immature permanent teeth and younger patients. Concomitant luxation injuries can negatively impact healing by disrupting vascular supply [27, 28]. Diagnosis requires assessment and evaluation of multiple clinical and radiographic indicators. Protection of the pulp is recommended by covering the exposed dentine tubules in uncomplicated crown fractures. Partial pulpotomies shows high success rates in traumatised permanent incisors with pulp exposure. DPC had lower success than partial pulpotomy, and although it may be suitable using CSCs in select cases [29], it is less practical than partial pulpotomy due to need for retention of the restoration. While pulp preservation is the goal for vital pulp therapy following traumatic injury, failure can occur resulting in pulp necrosis.

Immediate appropriate management of traumatic dental injuries can limit complications; however, avulsion and severe luxation injuries have higher complication rates. For traumatised immature permanent teeth with pulp necrosis, both regenerative endodontic therapy and apexification (CSC apical plug technique) are viable options [30, 31, 32, 33]. The protocols differentiate between the management of complications for primary and permanent teeth—while both may experience similar complications (pulp canal obliteration, root resorption), the management approaches differ significantly [28, 34, 35]. For permanent teeth, there is greater emphasis on preservation techniques like regenerative endodontic treatments, while primary teeth management focuses more on monitoring and intervention timing, which could be extraction or pulp therapy as indicated. External replacement resorption, which manifests clinically as ankylosis, is a progressive condition that cannot be halted or reversed. For young permanent teeth with unfavourable prognosis, alveolar preservation techniques (such as decoronation, or root submergence, or autotransplantation) or extraction are recommended [36, 37, 38, 39].

An overarching theme of the workgroup's translation of current best evidence into practice is the shift towards more conservative, pulp‐preserving approaches across all scenarios, with particular attention to the tooth's developmental stage and pulp status rather than just the extent of pathology. The recommendations also consistently emphasise the importance of careful case selection, pulp protection with CSC, and definitive restoration for treatment success. The emergence of CSCs and development of evidence‐based protocols has demonstrated that teeth previously considered as candidates for pulpectomy may be successfully managed with vital pulp therapy, even in cases of extremely deep caries and symptomatic irreversible pulpitis.

Current best evidence also supports more conservative approaches to caries removal. Complete caries removal is no longer considered appropriate in all cases, particularly when remnant caries, radiographically viewed as dentine separating caries from the pulp, can be sealed from the oral environment. Contemporary science suggests creating optimal conditions for pulp tissue repair through proper case selection, aseptic techniques, use of CSCs, and immediate definitive restoration. Evidence‐based practice relies not only on evidence but also on clinicians' skill and patients' preferences. Therefore, treatment options should be presented to patients/caregivers to facilitate a shared decision‐making process in developing treatment plans for pulp therapy.
